# Interaction between Connexin 43 and nitric oxide synthase in mice heart mitochondria

**DOI:** 10.1111/jcmm.12499

**Published:** 2015-02-09

**Authors:** Mücella Kirca, Petra Kleinbongard, Daniel Soetkamp, Jacqueline Heger, Csaba Csonka, Péter Ferdinandy, Rainer Schulz

**Affiliations:** a Physiologisches Institut, Justus-Liebig-UniversitätGiessen, Germany; b Institute for Pathophysiology, West German Heart and Vascular Center, University Schhool of Medicine EssenEssen, Germany; c Cardiovascular Research Group, Department of Biochemistry, University of SzegedSzeged, Hungary; d Pharmahungary GroupSzeged, Hungary; e Department of Pharmacology and Pharmacotherapy, Semmelweis UniversityBudapest, Hungary

**Keywords:** connexin, nitric oxide, heart, mitochondria

## Abstract

Connexin 43 (Cx43), which is highly expressed in the heart and especially in cardiomyocytes, interferes with the expression of nitric oxide synthase (NOS) isoforms. Conversely, Cx43 gene expression is down-regulated by nitric oxide derived from the inducible NOS. Thus, a complex interplay between Cx43 and NOS expression appears to exist. As cardiac mitochondria are supposed to contain a NOS, we now investigated the expression of NOS isoforms and the nitric oxide production rate in isolated mitochondria of wild-type and Cx43-deficient (Cx43^Cre-ER(T)/fl^) mice hearts. Mitochondria were isolated from hearts using differential centrifugation and purified *via* Percoll gradient ultracentrifugation. Isolated mitochondria were stained with an antibody against the mitochondrial marker protein adenine-nucleotide-translocator (ANT) in combination with either a neuronal NOS (nNOS) or an inducible NOS (iNOS) antibody and analysed using confocal laser scanning microscopy. The nitric oxide formation was quantified in purified mitochondria using the oxyhaemoglobin assay. Co-localization of predominantly nNOS (nNOS: 93 ± 4.1%; iNOS: 24.6 ± 7.5%) with ANT was detected in isolated mitochondria of wild-type mice. In contrast, iNOS expression was increased in Cx43^Cre-ER(T)/fl^ mitochondria (iNOS: 90.7 ± 3.2%; nNOS: 53.8 ± 17.5%). The mitochondrial nitric oxide formation was reduced in Cx43^Cre-ER(T)/fl^ mitochondria (0.14 ± 0.02 nmol/min./mg protein) in comparison to wild-type mitochondria (0.24 ± 0.02 nmol/min./mg). These are the first data demonstrating, that a reduced mitochondrial Cx43 content is associated with a switch of the mitochondrial NOS isoform and the respective mitochondrial rate of nitric oxide formation.

## Introduction

Connexin 43 (Cx43) is a gap junction protein, which is composed of four transmembrane domains, two extracellular and one intracellular loop, as well as cytosolic amino- and carboxy-termini. Cx43 is the target of different kinases and several phosphorylation sites are located within the carboxy-terminus. As the predominant protein of ventricular gap junctions, Cx43 forms hemichannels at the plasma membrane and regulates electrical cell coupling via gap junctions in myocardium. Cx43 is also present at the inner membrane of cardiomyocyte subsarcolemmal mitochondria (SSM), but not in interfibrillar mitochondria (IFM) 1. Cx43 is involved in cardioprotective signalling pathways 2 and reduction of mitochondrial Cx43 abrogates cardioprotection by ischaemic preconditioning 3. Nitric oxide is involved in cardioprotective signalling pathways and plays a central role in cardioprotection by ischaemic preconditioning 2,4,5. In the myocardium, nitric oxide is produced by different nitric oxide synthase (NOS) enzymes: neuronal NOS (nNOS), endothelial NOS (eNOS), inducible NOS (iNOS) and mitochondrial NOS (mtNOS) 6–8. The nNOS, eNOS and mtNOS are calcium sensitive and constitutively active, while iNOS is calcium insensitive and inducible. Nitric oxide is involved in the regulation of myocardial contractility, which is enhanced by low concentrations 9,10 and depressed by high concentrations 11. The mtNOS-dependent nitric oxide serves as mediator in the regulation of the energy metabolism, whereas it is notably associated with complex I (NADH dehydrogenase) and complex IV (cytochrome c oxidase) of the respiratory chain 12. In isolated mitochondria, mtNOS-dependent nitric oxide reversibly inhibits the cytochrome c oxidase 13,14, reduces the mitochondrial oxygen consumption and consequently ATP synthesis 15. After myocardial infarction, the expression, the activity and the localization of nNOS is changed 16–18. In cardiomyocytes, sarcoplasmatic reticulum localized nNOS translocates to the sarcolemma and into mitochondria 19,20.

The activated cardioprotective nitric oxide signalling pathway results in increased production of S-nitrosothiols (SNO). The S-nitrosylation is a modification of protein sulfhydryl residues by nitric oxide. This reaction changes the structure and activity of mitochondrial proteins in cardiomyocytes and protects proteins against irreversible oxidative injury during reperfusion 21,22. One of these proteins is mitochondrial complex I: the reversible inhibition of mitochondrial complex I by S-nitrosylation is cardioprotective by limiting reactive oxygen species (ROS) formation during reperfusion 23–25. Reduction of NOS-dependent nitric oxide formation through NOS inhibition abolishes the cardioprotection 26–28.

As yet, an interaction between nitric oxide and Cx43 is only described at the cellular level. Gene expression of Cx43 is down-regulated by iNOS-mediated nitric oxide formation 29–31. Conversely, a decreased nNOS expression by a reduction in Cx43 expression was demonstrated in mice bladder cells 32, in cultured cardiomyocytes 33 and astrocytes 34. How in detail Cx43 expression alters nNOS expression remains to be established, but the carboxy-terminus of Cx43 functions as transcription factor 35 and thus might bind to the promoter region of the nNOS gene. Interactions between Cx43 and nitric oxide in mitochondria are still completely unclear.

Therefore, the aim of this study was to investigate a Cx43-dependent alteration of NOS-mediated nitric oxide formation in cardiac mitochondria. As mitochondria are supposed to contain a NOS, an important way by which more insight into this mechanism of action could be obtained, is investigation of mitochondrial NOS isoform expression and measurement of mitochondrial nitric oxide formation rate in isolated mitochondria of wild-type and Cx43-deficient (Cx43^Cre-ER(T)/fl^) mouse hearts.

## Materials and methods

### Animal model

The present study was in accordance with the German laws for animal welfare and approved by the local review committee. It conforms to the *Guide for the Care and Use of Laboratory Animals* published by the US National Institutes of Health (NIH publication No. 85-23, revised 1996).

For experiments, 12–24-week-old male C57BL/6J wild-type (Charles River Laboratories) and heterozygous Cx43^Cre-ER(T)/fl^ mice (B6.129-*Gja1tm1Kdr,* JAX mice; Bar Harbor, ME) were used. Heterozygous Cx43^Cre-ER(T)/fl^ mice have the same phenotype as wild-type mice. The heterozygous knockout mice for Cx43 were generated by replacing exon-2 of the Cx43 gene by neomycin resistance gene 36. The Cx43 expression in mitochondria was characterized by Western blot. Cx43^Cre-ER(T)/fl^ mice showed lower mitochondrial Cx43 levels than wild-type mice (Fig.2A and B). As negative control served nNOS^−/−^ mice, which were provided by Dr. Martin Szibor from Bad Nauheim, Germany as a gift. The right ventricles were used as positive controls in Western blot analyses. Left ventricles (LV) were used for the isolation of mitochondria.

### Mitochondrial isolation

The mouse hearts were rapidly removed after cervical dislocation and SSM were isolated from ventricles as previously described 37 using a modification of the protocol described by Holmuhamedov *et al*. 38 and IFM were isolated as previously described 1 according to a modified protocol by Judge *et al*. 39. Isolated SSM were used for immunocytochemical analyses. For Western blot analysis and nitric oxide measurements, SSM and IFM were further purified by Percoll gradient (30%) ultracentrifugation (35,000 × g, 30 min.) for enrichment of mitochondrial proteins and elimination of other cellular elements. The protein concentration of the supernatant was determined using the Dc protein assay (Bio-Rad, Hercules, CA, USA).

To rule out putative mitochondrial contamination with NOS isoforms attached to the outer membrane of mitochondria, mitoplasts were prepared by removing the outer membrane using the digitonin detergent (2 mmol/l). The difference between mitochondria and mitoplasts was shown by the content of voltage-dependent anion channel (VDAC), a marker for the outer mitochondrial membrane. The presence of ATP-synthase α, manganese superoxide dismutase (MnSOD) and Cx43 proved the intactness of mitoplasts.

### Western blot analysis

For Western blot analysis, 100 μg of mitochondrial proteins were electrophoretically separated on 10% SDS-PAGE and transferred to nitrocellulose membranes. After blocking, the membranes were incubated with the following antibodies: rabbit polyclonal anti-rat Cx43 (Invitrogen, Carlsbad, CA, USA), ATP-synthase α (BD Transduction, San Jose, CA, USA), rabbit polyclonal anti-human MnSOD (Upstate, Lake Placid, NY, USA), rabbit polyclonal anti-human VDAC (Abcam, Cambridge, UK) or mouse monoclonal anti-rabbit Na^+^/K^+^-ATPase (Upstate), mouse monoclonal anti-dog sarcoplasmatic calcium (SERCA2)-ATPase (Sigma-Aldrich, Saint Louis, MO, USA). Immunoreactive signals were detected by chemiluminescence (SuperSignal West Femto Maximum Sensitivity Substrate, Pierce, Rockford, Il, USA) and quantified with the Scion Image software (Frederick, ML, USA).

### Immunocytochemistry

For immunocytochemistry, purified SSM were incubated simultaneously with the following primary antibodies: anti-Cx43 (rabbit polyclonal anti-rat Cx43, Zytomed, Berlin, Germany) with anti-cyclophilin D (mouse monoclonal anti-rat, MitoSciences, Eugene, OR, USA), anti-nNOS (BD Transduction) or anti-iNOS (BD Transduction) with anti-ANT (Santa Cruz, Heidelberg, Germany) respectively. After washing, SSM were incubated with corresponding secondary antibodies (TRITC donkey anti-rabbit and FITC goat anti-rabbit for Cx43, TRITC donkey anti-rabbit and FITC goat antimouse for nNOS, TRITC bovine antimouse and FITC bovine anti-rabbit for iNOS). SSM were examined by confocal laser scan microscopy (Pascal, Zeiss, Jena, Germany, or Leica TCSSP2AOBS, Wetzlar, Germany) at 630 × magnification.

### Measurement of cardiac nitric oxide

#### Oxyhaemoglobin assay

The nitric oxide formation in SSM (0.86 mg/ml) of wild-type mouse hearts was measured by the oxyhaemoglobin assay. The oxyhaemoglobin assay was modified to Feelisch *et al*. 40. The nitric oxide formation was evaluated as the change in the absorbance at 401 and 590 nm using a diode array spectrophotometer (HP 8453; Hewlett Packard, Waldbronn, Germany). SSM, IFM or mitoplasts were incubated in incubation buffer (pH 7.4) with L-arginine (1 mmol/l), BH_4_ (10 μmol/l), CaCl_2_ (100 μmol/l) and oxyhaemoglobin (HbO_2_, 50 μmol/l) for 60 min. at 37°C. SOD (1 U/ml) and catalase (50 U/ml) were also present in the reaction medium to avoid unspecific reactions. SOD catalysed superoxide to oxygen and hydrogen peroxide. Catalase dissipated the hydrogen peroxide in oxygen and water.

Every 10 min., an aliquot was diluted in isolation buffer (pH 7.4) and the oxidation of HbO_2_ to methaemoglobin by nitric oxide was measured. The nitric oxide scavenger carboxy-PTIO (2-(4-Carboxyphenyl)-4,4,5,5-tetramethylimidazoline-1-oxyl-3-oxide) was used to determine the specificity of the nitric oxide signal. NOS-dependent nitric oxide formation was confirmed by the selective NOS inhibitors SMTC and 1400 W (selective for nNOS/iNOS) 41,42.

#### Electron spin resonance spectroscopy

Mitochondrial nitric oxide content was also measured using electron spin resonance spectroscopy (ESR) in isolated SSM. An aqueous spin trapping solution Fe^2+^–(MGD)_2_ was prepared before each experiment as described 4,43. GSNO was resolved in phosphate buffer (35 mg/ml) and used as positive control. Isolated SSM and GSNO (200 μmol/l) were treated with L-arginine (1 mmol/l), BH_4_ (10 μmol/l), CaCl_2_ (100 μmol/l), SOD (1 U/ml) and catalase (50 U/ml) and Fe^2+^–(MGD)_2_ and incubated for 10 min. at 37°C. The prepared samples were then placed into quartz ESR tubes and frozen immediately in liquid nitrogen. Samples were assayed for ESR spectra of the relatively stable nitric oxide–Fe^2+^–(MGD)_2_ adduct. ESR spectra were recorded with a ECS106 spectrometer (Bruker, Rheinstetten, Germany) operating at X band with 100 kHz modulation frequency at a temperature of 160 K, using 10 mW microwave power to avoid saturation. Scans were traced with 2.85 G modulation amplitude, 340 G sweep width, and 3356 G central field as described 44,45. Analysis of nitric oxide content was performed with double integration of the nitric oxide signal as described previously 46.

### Statistics

Data are reported as mean ± SEM. Confocal laser scanning microscopy data for Cx43, nNOS and iNOS were compared by two-way anova, subsequent Bonferroni test. Quantitative Western blot data for Cx43 and ATP-synthase α were compared by one-way anova. The nitric oxide formation by the oxyhaemoglobin assay and the ESR spectroscopy in SSM of wild-type, Cx43^Cre-ER(T)/fl^ and nNOS^−/−^ mice was compared by one-way anova respectively. A *P* < 0.05 was considered to indicate a significant difference.

## Results

### NOS expression in SSM of Cx43-deficient mice

**Fig 1:**
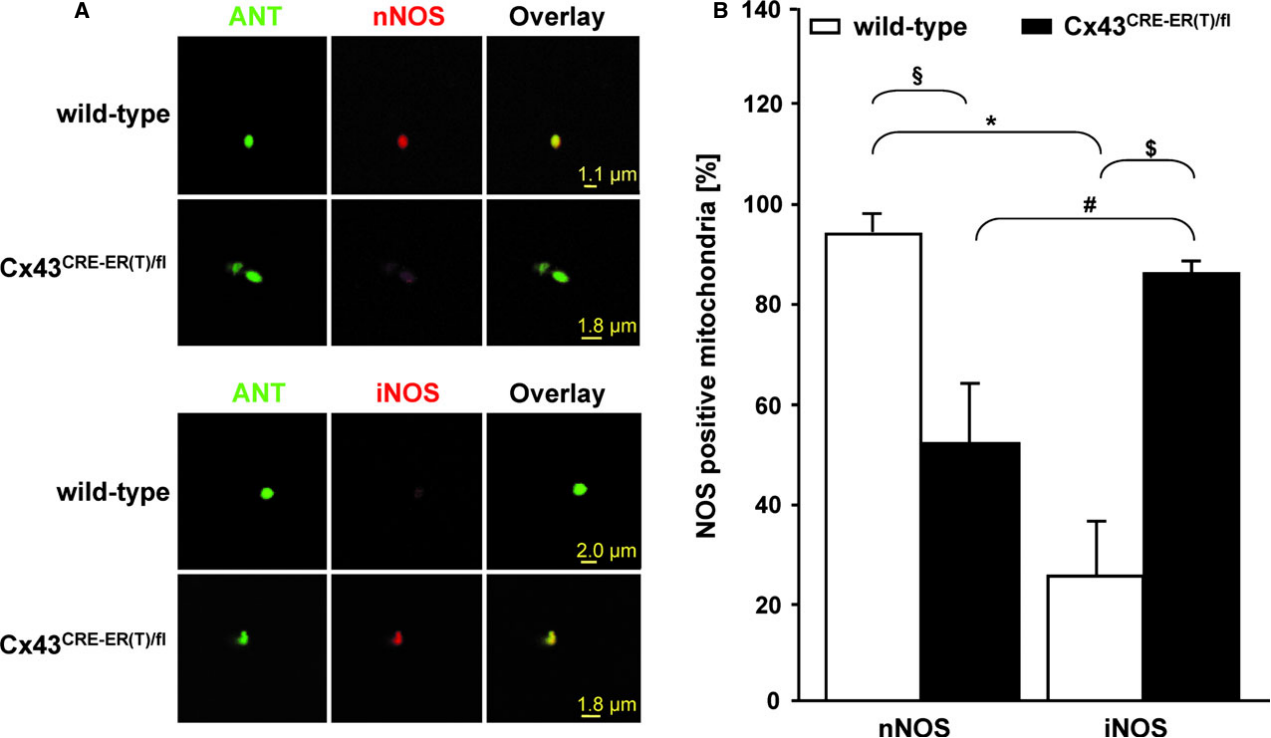
Mitochondrial NOS expression in subsarcolemmal mitochondria of wild-type mice and Cx43^Cre-ER(T)/fl^ mice. (A) Subsarcolemmal mitochondria (SSM) isolated from the ventricles of Cx43^Cre-ER(T)/fl^ and wild-type mice were stained with antibodies against nNOS or iNOS (red) and the mitochondrial marker cytochrome c (green) and analysed by confocal laser scanning microscopy. Merged image shows co-localization pixels in yellow. (B) Amount in per cent of nNOS- and iNOS-positive SSM were referred to 100%. Individual preparations of *n* = 7. ^§^*P* < 0.003 nNOS of wild-type *versus* nNOS of Cx43^Cre-ER(T)/fl^, *P < 0.001 nNOS of wild-type *versus* iNOS of wild-type, ^#^*P* < 0.02 iNOS of Cx43^Cre-ER(T)/fl^ *versus* nNOS of Cx43^Cre-ER(T)/fl^, ^$^*P* < 0.002 iNOS of Cx43^Cre-ER(T)/fl^ *versus* iNOS of wild-type.

Confocal laser scanning microscopy demonstrated mainly nNOS immunoreactivity and a weak expression of iNOS in isolated SSM from wild-type mice (Fig.1A). Conversely, in SSM of Cx43^Cre-ER(T)/fl^ mice, iNOS immunoreactivity was increased while nNOS expression was reduced (Fig.1A). As marker for mitochondria, the inner mitochondrial membrane protein ANT was detected. The statistical evaluation of NOS expression showed a significant difference between mitochondria expressing nNOS and iNOS in wild-type mice (93 ± 4.1% *versus* 24.6 ± 7.5% co-localization of NOS with ANT, *n* = 7 individual preparations, Fig.1B). The nNOS expression in SSM of Cx43^Cre-ER(T)/fl^ mice (53.8 ± 17.5% co-localization of NOS with ANT, *n* = 7 individual preparations) was also significantly reduced compared to wild-type mice. In contrast, the iNOS expression (90.7 ± 3.2% co-localization of NOS with ANT, *n* = 7 individual preparations, Fig.1B) in SSM of Cx43^Cre-ER(T)/fl^ mice was significantly increased compared to iNOS in wild-type mice.

**Fig 2:**
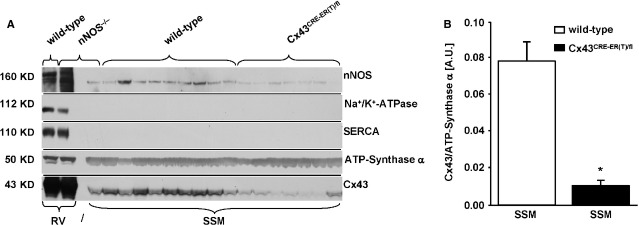
Expression of nNOS in subsarcolemmal mitochondria. (A) The expression of nNOS is presented in isolated subsarcolemmal mitochondria (SSM) of Cx43^Cre-ER(T)/fl^ (*n* = 9) and wild-type mice (*n* = 7). Right ventricles (RV) from wild-type and nNOS^−/−^ mice served as positive and negative control. Na^+^/K^+^-ATPase and SERCA served for the purity of the mitochondrial preparation. The Cx43 content showed the difference between the two mice strains. (B) The statistical evaluation of the Cx43 signal in SSM as related to the ATP-synthase α signal is present as mean ± SEM (* significance with *P* < 0.05 between wild-type and Cx43^Cre-ER(T)/fl^ mice).

To verify the immunocytochemical results by Western blot analysis in the mitochondrial samples of wild-type and Cx43^Cre-ER(T)/fl^ mice, immunoblotting with anti-nNOS antibody against the amino-terminus showed no distinctive band at 160 kD compared to the positive control (right ventricle, Fig.2A). Only an unspecific band at 140 kD, which was also seen in mitochondria of nNOS^−/−^ mice (negative control), was present (Fig.2A). Antibodies against the iNOS isoform showed no visible band. Mitochondria were not contaminated with proteins of sarcolemma and with sarcoplasmatic reticulum as shown by the absence of Na^+^/K^+^-ATPase and SERCA immunoreactivity (Fig.2A). Cx43 protein content was normalized to mitochondrial marker protein ATP-synthase α (Fig.2B). Immunoprecipitation analysis also showed no detectable signal of the NOS isoforms. By definition, mitochondrial Cx43 expression in Cx43^Cre-ER(T)/fl^ mice was significantly reduced compared to wild-type mice.

### Nitric oxide formation in Cx43-deficient mice

**Fig 3:**
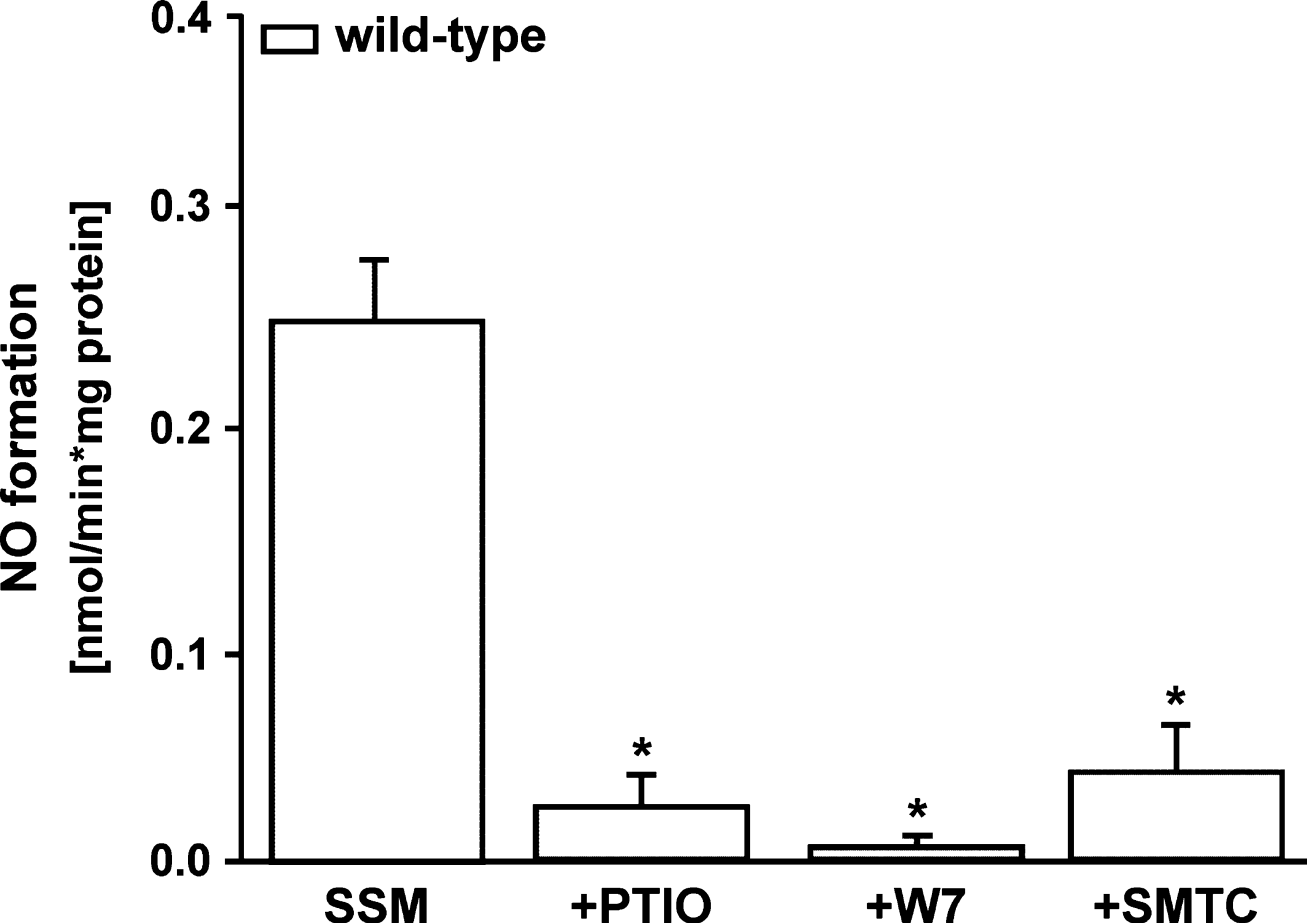
Basal nitric oxide formation in subsarcolemmal mitochondria of wild-type mice. PTIO (*n* = 7) reduced the nitric oxide formation. The enzymatic NOS inhibition by the inhibitors W7 (non-selective, *n* = 5) and SMTC (nNOS selective, *n* = 7) reduced nitric oxide formation. *P < 0.001 indicates significant difference after treatment with PTIO, W7 or SMTC. Wild-type mice (*n* = 13).

Nitric oxide formation was measured by the oxyhaemoglobin assay in SSM of wild-type mice (Fig.3). The basal NOS activity resulted in a nitric oxide formation of 0.24 ± 0.02 nmol/min./mg protein (*n* = 15). The specificity of the nitric oxide signal was shown by the nitric oxide scavenger PTIO. Inhibition of nNOS using the non-selective (W7) or the selective nNOS inhibitor (SMTC) resulted in a significant reduction of the mitochondrial nitric oxide formation.

**Fig 4:**
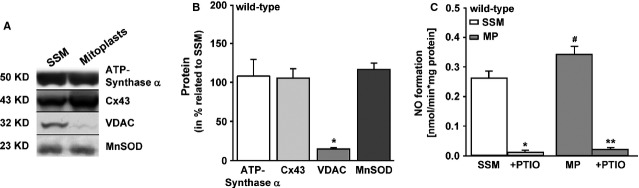
Nitric oxide formation in mitoplasts of wild-type mice. (A) Representative Western blot shows the difference between subsarcolemmal mitochondria (SSM, *n* = 5) and mitoplast (MP, *n* = 6) preparation by the absence of the outer mitochondrial membrane protein voltage-dependent anion channel (VDAC). ^#^*P* < 0.05 indicates the significant difference between MP and mitochondria. (B) Statistical evaluation presents the mean ± SEM of MP as related to SSM (in %), * significance with *P* < 0.05 MP and mitochondria. (C) Nitric oxide formation in MP (*n* = 11) and mitochondria (*n* = 10). PTIO scavenged the accumulated nitric oxide. * Significance between SSM ± PTIO (*P* < 0.001), ** significance between MP ± PTIO (*P* < 0.001).

Digitonin treatment of mitochondria significantly reduced the content of the outer mitochondrial membrane protein VDAC to 14 ± 2.6% (*n* = 6) of the signal of untreated mitochondria (set as 100%, Fig.4A). The unchanged level of ATP-synthase α (93 ± 27% protein content of mitoplasts compared to mitochondria set as 100%, *n* = 6), MnSOD (116 ± 18%, *n* = 6) and mitochondrial Cx43 (105 ± 27%, *n* = 6) confirmed an intact inner membrane of mitoplasts (Fig.4B). The nitric oxide production in mitoplasts was comparable with the nitric oxide production in SSM of wild-type mice (Fig.4C). Therefore, a contamination of mitochondria with cellular NOS isoforms attached to the outer mitochondrial membrane as explanation for the measured nitric oxide formation appeared unlikely and an existence of nNOS-dependent mitochondrial nitric oxide production was confirmed. Again the nitric oxide specificity of the signal was shown by PTIO.

**Fig 5:**
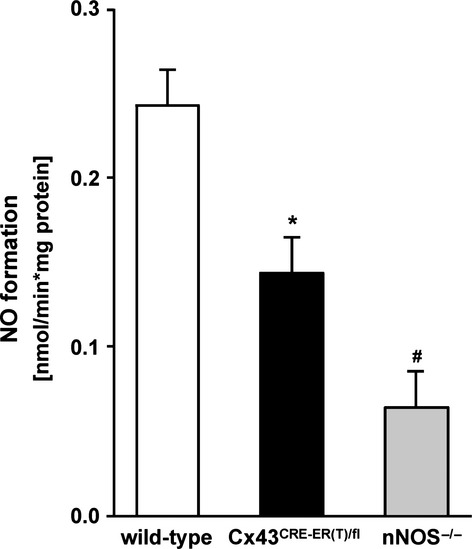
Mitochondrial nitric oxide formation and connexin 43 deficiency. Comparison of nitric oxide formation in subsarcolemmal mitochondria (SSM) of wild-type (*n* = 15) and Cx43^Cre-ER(T)/fl^ mice (*n* = 12, *P < 0.001). NNOS^−/−^ mice served as negative controls (*n* = 4). # indicates the significant difference between wild-type and nNOS^−/−^ mice (*P* < 0.001).

**Fig 6:**
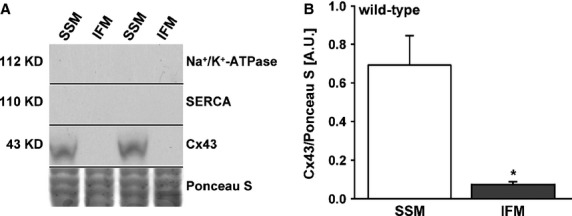
Content of connexin 43 in subsarcolemmal and interfibrillar mitochondria. (A) Representative Western blot of Cx43 in isolated subsarcolemmal (SSM, *n* = 5) and interfibrillar (IFM, *n* = 5) mitochondria from left ventricles (LV). SERCA and Na^+^/K^+^-ATPase served as marker protein for the purity of the mitochondrial preparation. Ponceau S staining shows equal protein loading. (B) Quantification of the Cx43 content in SSM and IFM normalized to Ponceau S staining (*n* = 5 individual preparations, *P < 0.001).

The NOS-dependent nitric oxide formation in Cx43^Cre-ER(T)/fl^ mice (0.14 ± 0.02 nmol/min./mg, *n* = 12) was reduced in comparison to wild-type mice (0.24 ± 0.02 nmol/min./mg; Fig.5). Inhibition of nNOS by SMTC in SSM of wild-type mice strongly reduced the nitric oxide formation (0.05 ± 0.01 nmol/min./mg; Table1). SMTC showed a lower effect of reducing of nitric oxide formation in SSM of Cx43^Cre-ER(T)/fl^ mice (0.06 ± 0.02 nmol/min./mg). In contrast, 1400 W reduced the nitric oxide formation in SSM of Cx43^Cre-ER(T)/fl^ to 62%. A similar result was detectable by iNOS inhibition in SSM of wild-type mice (58% nitric oxide formation). The negative control showed a residual NOS activity of 0.06 ± 0.02 nmol/min./mg (*n* = 4). The enzymatic residual NOS activity was completely reduced by SMTC and 1400 W. To confirm the Cx43 dependence of the NOS shift, we used isolated IFM of wild-type mice, the mitochondrial subpopulation without Cx43, as internal control 1. The purity of IFM preparation was controlled by Western blot analysis. IFM were not contaminated with proteins of sarcolemma and sarcoplasmatic reticulum as shown by the absence of Na^+^/K^+^-ATPase and SERCA immunoreactivity (Fig.6A). Cx43 was also detected exclusively in SSM (Fig.6B).

**Table 1:** Nitric oxide formation after nitric oxide synthase inhibition in all mice strains

Mice strains	Nitric oxide formation (nmol/min./mg)		
	Basal	After nNOS inhibition (SMTC)	After iNOS inhibition (1400 W)
Wild-type	0.24 ± 0.02	0.05 ± 0.01*	0.13 ± 0.01*
Cx43^Cre-ER(T)/fl^	0.14 ± 0.02	0.06 ± 0.01†	0.05 ± 0.02†
nNOS^−/−^	0.06 ± 0.02	0.03 ± 0.01‡	0.009 ± 0.009‡

* Wild-type ± SMTC (*P* < 0.001) and ±1400 W (*P* < 0.001).

† Cx43^Cre-ER(T)/fl^ ± SMTC (*P* < 0.001) and ±1400 W (*P* < 0.001).

‡ nNOS^−/−^ mice ± SMTC (*P* < 0.005) and ±1400 W (*P* < 0.001).

SMTC, S-methyl-thiocitrulline, iNOS, inducible nitric oxide synthase; nNOS, neuronal nitric oxide synthase.

**Fig 7:**
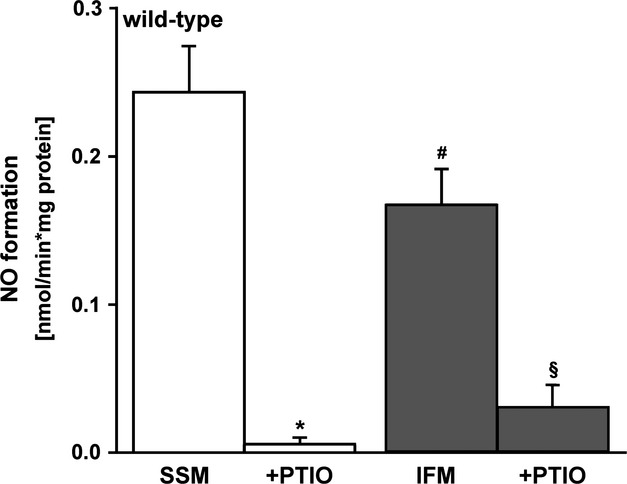
Nitric oxide formation in subsarcolemmal and interfibrillar mitochondria. Nitric oxide formation was compared between subsarcolemmal (SSM, *n* = 10 individual experiments) and interfibrillar mitochondria (IFM, *n* = 9 individual experiments) of wild-type mice (^#^*P* < 0.04 between SSM and IFM). Isolated IFM served as internal control. Nitric oxide specificity was shown by PTIO. *P < 0.001 SSM ± PTIO, ^§^*P* < 0.001 IFM ± PTIO.

Interfibrillar mitochondria showed a significantly reduced nitric oxide formation (0.17 ± 0.02 nmol/min./mg, *n* = 9) in contrast to SSM (0.24 ± 0.03 nmol/min./mg, *n* = 10; Fig.7). In presence of the nitric oxide scavenger PTIO, the nitric oxide formation was almost undetectable in both mitochondrial preparations (0.007 ± 0.006 nmol/min./mg in SSM; 0.03 ± 0.01 nmol/min./mg IFM; Fig.7).

**Fig 8:**
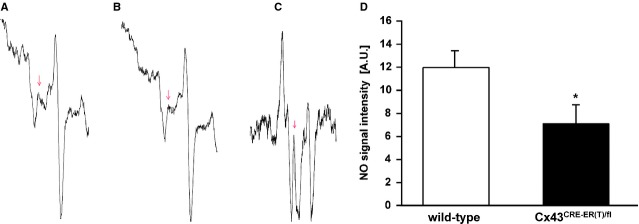
Formation of nitric oxide by electron spin resonance spectroscopy after spin trapping with Fe^2+^–MGD complex. Representative electron spin resonance (ESR) spectra of nitric oxide–Fe^2+^–MGD adduct in SSM of (A) wild-type and (B) Cx43^Cre-ER(T)/fl^ mice. (C) GSNO served as positive control. ESR parameters: X band, 100 kHz modulation frequency, 160 K, 10 mW microwave power, 2.85 G modulation amplitude, 340 G sweep width, and 3356 G central field. (D) The nitric oxide content in subsarcolemmal mitochondria (in arbitrary units) obtained from the relative signal amplitude of the nitric oxide–Fe^2+^–MGD complex in peak three of wild-type and Cx43^Cre-ER(T)/fl^ mice (*n* = 6 preparations, *P < 0.04).

Another independent method for quantifying nitric oxide formation is the ESR technique. The ESR spectrum of the positive control (GSNO) showed a prominent nitric oxide–Fe^2+^–MGD triplet (Fig.8A). In the SSM of wild-type mice, a basal spectrum of nitric oxide–Fe^2+^–MGD triplet was detected (Fig.8B). In the SSM of Cx43^Cre-ER(T)/fl^ mice, the intensity of the specific spectra of nitric oxide–Fe^2+^–MGD complex was reduced compared to the wild-type mice (Fig.8C). Peak three is an indicator of the nitric oxide content in SSM of wild-type and Cx43^Cre-ER(T)/fl^ mice obtained from relative signal amplitudes of nitric oxide–Fe^2+^–MGD complex. The nitric oxide content of peak three in SSM of Cx43^Cre-ER(T)/fl^ mice was significantly reduced compared to wild-type mice (*n* = 6 preparations, Fig.8D).

## Discussion

In the present study, Cx43 expression correlated with the NOS isoform mainly present in SSM. While in SSM from wild-type hearts nNOS was the main isoform, in SSM from Cx43-deficient mice hearts iNOS dominated.

The constitutive mitochondrial NOS isoform expressed differs among species. The iNOS was present in porcine and rat heart mitochondria 47,48, while nNOS was mainly found to be expressed in mitochondria from mice hearts 49. The latter finding was confirmed in the present study in SSM isolated from wild-type C57BL/6J mice.

In previous studies, also eNOS protein was localized to mitochondria 50; however, in the present study while we could detect an eNOS signal in the vascular endothelium, there was no signal detected in mitochondria (data not shown). More recently, increased translocation of eNOS protein (located in caveolae) to the outer mitochondrial membrane was demonstrated upon short periods of ischaemia/reperfusion 51. Thus, some of the differences observed in mitochondrial eNOS expression might relate to the presence or absence of attached sarcolemmal proteins which was excluded in our mitochondrial preparation.

The mtNOS protein was mainly detected by immunohistochemical 8,52 and biochemical methods 48,53–55. Similarly, the presence of mtNOS in the present study could only be confirmed by immunohistochemistry and direct measurement of nitric oxide formation from isolated mitochondria; no specific NOS signal was detected by Western blot using commercially available antibodies. One explanation for the lack of detection of NOS expression in the Western blot analysis is the limited sensitivity (1/10) in comparison to laser scan microscopy 56. This assumption is supported by the fact that with massive overexpression of nNOS in mice, a mitochondrial signal was detected also by Western blot 57.

Given the lack of NOS detection in mitochondria by a method like Western blot, the existence of NOS in mitochondria is still under discussion 58. In different studies, mitochondrial NOS was attributed to a contamination of the preparation or the attachment of NOS to the outer mitochondrial membrane 59–61. To circumvent this problem, we generated mitoplasts 1 and the rate of nitric oxide formation of mitoplasts was similar to that of intact SSM.

An array of methods exists for the detection of mtNOS-dependent nitric oxide formation including photometrical (oxyhaemoglobin assay, DAF-2 assay) 58,62–64, electrochemical (porphyrinic microsensor) 49 and radioactive (citrulline assay) techniques 7. The sensitivity and specificity of the oxyhaemoglobin assay is sufficient to detect mtNOS-dependent changes in nitric oxide formation 48,49,58,65 and allows real-time detection of generated nitric oxide 66. In the present study, we were able to confirm the rate of NOS-derived basal nitric oxide formation in the SSM from wild-type hearts. The nitric oxide formation of about 0.24 nmol/min. mg and was thus close to the range of NOS-derived nitric oxide formation rates from rat heart mitochondria (0.37 nmol/min./mg and 0.69 nmol/min./mg) 48,65,67. Composition of the buffer, the temperature and the mitochondrial metabolic state could cause the small differences in basal nitric oxide formation. However, the specificity of the detected nitric oxide signal was proven by scavenging nitric oxide through addition of PTIO. The strong reduction of the nitric oxide formation by selective nNOS inhibition confirmed a mainly expressed mitochondrial nNOS in wild-type mice. The slight reduction of the nitric oxide formation by the selective iNOS inhibitor approved a small amount of active mitochondrial iNOS in wild-type mice.

Concomitant with an enhanced mitochondrial iNOS expression, an increased nitric oxide formation rate was expected. However, and to our surprise, the enhanced mitochondrial iNOS and reduced mitochondrial nNOS expression in Cx43-deficient hearts was associated with a reduced rate of nitric oxide formation in mitochondria derived from Cx43-deficient mice hearts. These data from the oxyhaemoglobin assay were confirmed by a second independent technique, namely ESR, which also shows a high nitric oxide sensitivity 68.

Indeed, iNOS may be present in two forms either as uncoupled monomer and/or coupled dimer. Zhang *et al*., found in a stress-induced myocardium of wild-type mice, both iNOS monomer and iNOS dimer 69. One can speculate that the reduced nitric oxide formation in mitochondria from Cx43-deficient mice relates to the presence of an enhanced monomer iNOS isoform and coincident low dimer iNOS isoform.

Mitochondrial nitric oxide formation in nNOS^−/−^ mice (negative control) was because of an enzymatic residual activity of the conceivably available splice variants nNOS-β und nNOS-γ 70,71, which were inhibited to a great extent by the selective nNOS inhibitor, and some contribution of iNOS. The remaining nitric oxide formation might represent non-enzymatically released nitric oxide from nitrite 72, which have been suggested to contribute to the cardioprotective effect of nitrite 73,74.

Nitrosylation of several respiratory complexes including complex I are supposed to impair respiration 25. Indeed, IFM which have a lower nitric oxide production as compared to SSM have a higher respiratory rate. Although SSM of Cx43-deficient mice also have a reduced nitric oxide production as compared to SSM of wild-type mice, they nevertheless have a reduced respiratory rate and ATP production 75. One potential explanation for this contradictory finding might relate to decreased potassium flux in Cx43-deficient mice upon ADP stimulation, which impairs matrix volume recovery and thus mitochondrial oxygen consumption 76.

**Fig 9:**
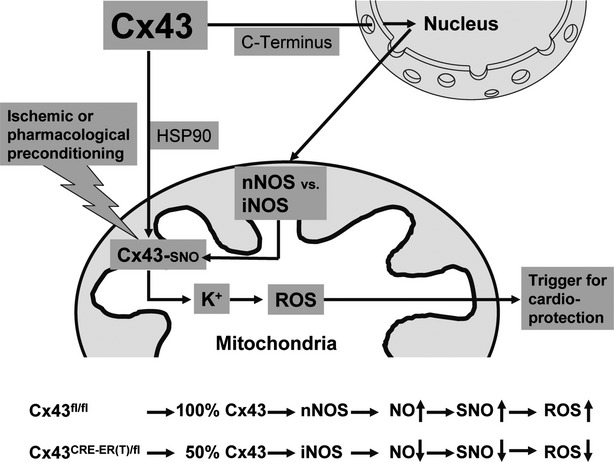
Scheme of nitric oxide and Connexin 43 (Cx43) in the context of cardioprotection. The mitochondrial NOS isoform expression and subsequently the mitochondrial nitric oxide production depends on the level of Cx43 expression. An overall reduction of Cx43 expression not only reduced mitochondrial Cx43 but also mitochondrial nNOS expression with a subsequent decrease in mitochondrial nitric oxide production and overall protein S-nitrosation, both contributing to a reduction of mitochondrial matrix potassium influx and reactive oxygen species formation upon an external stimulus such as ischaemic or pharmacological preconditioning, thereby contributing to the lack of endogenous cardioprotection seen in Cx43-deficient mice.

Indeed, we have recently shown that increased S-nitrosation of Cx43 enhanced potassium influx into the mitochondrial matrix as well as the generation of reactive oxygen species 77, the latter being an important signalling molecule for endogenous cardioprotection. Importantly, in the above study, ischaemic preconditioning increased S-nitrosation of mitochondrial Cx43. The present study adds one more facet to the interaction of nitric oxide and Cx43 in that the mtNOS isoform and subsequently the mitochondrial nitric oxide production depends on the level of Cx43 expression (Fig.9). An overall reduction of Cx43 expression in Cx43-deficient mice not only reduced mitochondrial Cx43 but also mitochondrial nNOS expression with a subsequent decrease in mitochondrial nitric oxide production and overall protein S-nitrosation (Data S1), both contributing to a reduction of mitochondrial matrix potassium influx and reactive oxygen species formation upon an external stimulus such as ischaemic or pharmacological preconditioning thereby contributing to the lack of endogenous cardioprotection seen in Cx43-deficient mice 78,79.

In conclusion, the present study showed that reduced mitochondrial Cx43 content is associated with a switch of the mitochondrial NOS isoform and the respective mitochondrial nitric oxide production rate.

## Supporting Information

**Data S1** Quantification of S-nitrosated protein of Cx43 in heart.
