# Supplementary material for: Interaction between Connexin 43 and nitric oxide synthase in mice heart mitochondria
Source: J Cell Mol Med. 2015 Feb 9;19(4):815–25. doi: 10.1111/jcmm.12499 (PMC4395196; doi:10.1111/jcmm.12499)
Supplement: Supplementary file 1 [file jcmm0019-0815-sd1.doc]

**Supplementary file**

**Methods**

**Labeling and precipitation of S-nitrosated modified proteins**

The amount of S-nitrosated protein was quantified in hearts from Cx43^Cre-ER(T)/fl^ and wild-type mice. The mice hearts were minced in cell lysis buffer supplemented with an EDTA-free complete protease inhibitor tablet (Roche Diagnostics, Indianapolis, IN, USA), phosphatase inhibitor (Roche Diagnostics, Grenzach, Germany or Indianapolis, IN, USA) and 0.1 mmol neocuproine. Non-lysed particles were spun down at 9.300 g for 10 min at 4°C and then a modified biotin switch method was used for labeling and quantification of SNO residues as previously described [1]. Protein samples (250 µg) were diluted in HEN buffer (250 mmol HEPES-NaOH pH 7.7, 1 mmol an EDTA, and 0.1 mmol neocuproine), supplemented with EDTA-free complete protease inhibitor tablet, phosphatase inhibitor (Roche Diagnostics, Grenzach, Germany or Indianapolis, IN, USA) and 2.5% SDS (wt/vol). To block free thiols, 50 mmol N-ethylmaleimide (NEM; Sigma-Aldrich, St. Louis, MO, USA or Heidenheim, Germany) was used. After incubation for 20 min at 50°C with gentle mixing every 5 min, free thiols were labeled with NEM and could not be modified. This procedure was stopped by removing NEM via cold acetone precipitation (-20°C). The samples were then resuspended in HEN buffer with 1% SDS (wt/vol) containing 1 mmol ascorbate (Sigma-Aldrich, St. Louis, MO, USA) for reduction of SNO modified cysteine residues. Reduced SNO groups were labeled with N-(biotinoyl)-N-(iodoacetyl)ethylenediamine (BIAM; Sigma-Aldrich, St. Louis, MO, USA or Heidenheim, Germany). Prior to incubating samples with streptavidin-agarose beads (Sigma-Aldrich, St. Louis, MO, USA) for precipitation of SNO modified proteins, 2 µl of loading control was taken. Precipitation was performed overnight with rotation at 4°C in the dark. Samples were washed three times with HEN buffer, eluted in 30 µl sample buffer with 10 M urea and heated at 95°C for 5 min. Silver staining for quantification of SNO modified proteins and Coomassie staining (loading control) was subsequently performed using 10% Bis-Tris-SDS-gels.

**ROS production**

ROS was measured according to the method recently described in detail [2]. One mg of freshly isolated mitochondria were added to 2 ml incubation buffer (150 mM KCl, 7 mM NaCl, 2 mM KH_2_PO_4_, 1 mM MgCl_2_, and 6 mM MOPS, pH 7.4) containing horse radish peroxidase (HRP) and Amplex UltraRed reagent (Life Technologies, Darmstadt, Germany). Amplex UltraRed is a fluorogenic substrate for HRP that reacts with hydrogen peroxide (H_2_O_2_). The measurement of ROS production was done in non-energized mitochondria for 10 min.


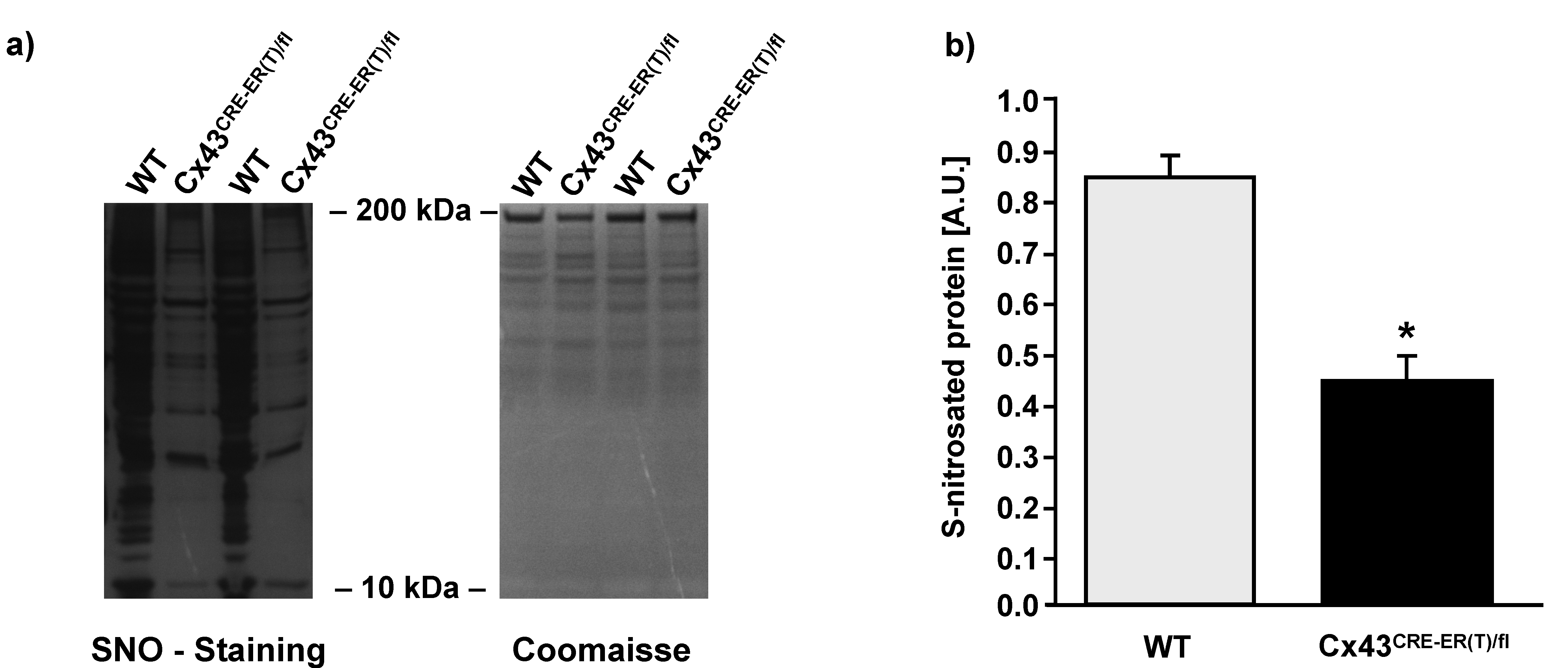


Supplemental figure 1 **Quantification of S-nitrosated protein of Cx43 in heart.** (a) The silver staining presented the reduced SNO modified proteins in right ventricles of Cx43^Cre-ER(T)/fl^ (n= 4) compare to wild-type mice (WT, n= 4). Coomassie-staining served as loading control. (b) The amount of S-nitrosated protein was quantified in hearts from wild-type mice Cx43^Cre-ER(T)/fl^. (significance with * p = 0.01 between WT and Cx43^Cre-ER(T)/fl^ mice).


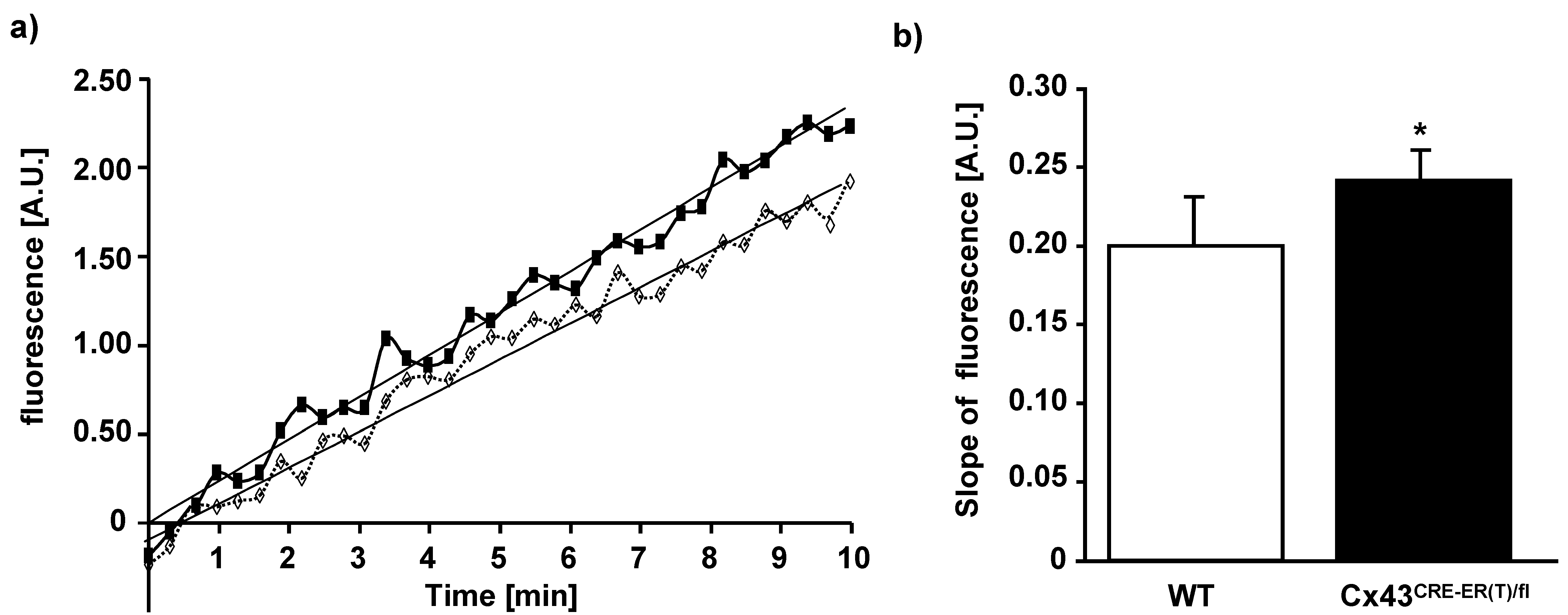


Supplemental figure 2 **Reactive oxygen species formation in mitochondria.** (a) Time-dependent measurement and (b) quantification of reactive oxygen species (ROS) in isolated mitochondria of Cx43^Cre-ER(T)/fl^ (n= 4) and wild-type (WT, n= 4) mice. * p = 0.054.

References

1. **Jaffrey SR, Snyder SH**. The biotin switch method for the detection of S-nitrosylated proteins. *Sci STKE*. 2001; 2001: l1.

2. **Soetkamp D, Nguyen TT, Menazza S, *et al.*** S-nitrosation of mitochondrial connexin 43 regulates mitochondrial function. *Basic Res Cardiol*. 2014; 109: 433.
